# The ratio of superoxide dismutase to standard deviation of erythrocyte distribution width as a predictor of systemic lupus erythematosus

**DOI:** 10.1002/jcla.23230

**Published:** 2020-02-29

**Authors:** Ling Yan, Bo Wang, Shizhi Chen, Hua Zhou, Pu Li, Lijing Zhou, Qing Zhao, Bo Wang, Weixian Chen

**Affiliations:** ^1^ Department of Clinical Laboratory The Second Affiliated Hospital of Chongqing Medical University Chongqing China

**Keywords:** predictor, ratio, standard deviation of erythrocyte distribution width, superoxide dismutase, systemic lupus erythematosus

## Abstract

**Background:**

To explore the clinical value of the serum superoxide dismutase‐to‐standard deviation of erythrocyte distribution width ratio (SRSR) in systemic lupus erythematosus (SLE).

**Methods:**

A total of 222 SLE patients from the Rheumatology and Immunology Department in the Second Affiliated Hospital of Chongqing Medical University from January 2017 to April 2019 were collected as the experimental group, and a total of 202 healthy physical examiners were extracted as the control group. Neutrophil‐to‐lymphocyte ratio (NLR), superoxide dismutase‐to‐standard deviation of erythrocyte distribution width ratio (SRSR), and platelet‐to‐lymphocyte ratio (PLR) were calculated from the collected data and then compared the level of the above three indexes between the two groups. In addition, we analyzed the association between SRSR and clinically relevant indicators.

**Results:**

We found that the SRSR of SLE patients was significantly lower than healthy control group, by analyzing the receiver operating characteristic (ROC) curve; it revealed that the SRSR had higher specificity and sensitivity than either superoxide dismutase (SOD) or standard deviation of erythrocyte distribution width (RDW‐SD) alone. The area under the curve (AUC) for SRSR was significantly larger than either SOD or RDW‐SD alone, and the AUC for SRSR was also larger than NLR and PLR. And it was found that SRSR was independently correlated with SLE disease activity through multiple linear regression analysis.

**Conclusion:**

SRSR is a useful biomarker for the diagnosis of SLE, and it is of great significance in the clinical application.

## 1 INTRODUCTION

Systemic lupus erythematosus is a chronic inflammatory autoimmune disease, and it can affect many organ systems. There are about 31‐70 SLE patients per 100 000 population in China.^1^ The disease is mainly in female with high disability rate and poor prognosis. The basic pathological change is the vasculitis induced by antigen‐antibody immune complex, and the pathogenesis is not completely clear. Studies have shown that the nosogenesis of SLE is related to the imbalance of the oxidation‐antioxidant system caused by increased peroxidation or reduced antioxidant capacity in vivo.^2^ Zhang qun et al showed that the content of serum malondialdehyde in SLE group was significantly increased and that the body of patients showed a peroxidation state. They also provided evidence that the total antioxidant capacity of serum was significantly reduced via comparing with the healthy people, suggesting that the antioxidant defense capacity of SLE patients was weakened.^3^

The body's antioxidant system is divided into enzymatic antioxidants and non‐enzymatic antioxidant substances. SOD as one of the important enzymatic antioxidant substances in the body can clean up oxygen free radicals contained in the body. Its activity level reflects the body's ability to scavenge free radicals. Furthermore, the level of SOD is a sensitive index in the body to play an antioxidant role. The RDW‐SD, which is a part of routine blood test, mainly refers to the degree of variation about the volume of red blood corpuscle in circulating plasma. Over the years, growing evidence has reported that RDW‐SD is associated with the inflammatory response in a variety of diseases. Currently, there is a lack of literature on the relationship between SOD‐to‐RDW‐SD ratio (SRSR) and SLE. In this research, according to analyzing the clinical data of 222 patients with SLE and 202 healthy people, we explored the possible relationship between SRSR and activity of SLE, and evaluated the predictive value of SRSR for inflammation in patients with SLE. In recent years, NLR and PLR have been reported to be new biomarkers obtained by ratio, which can reflect the inflammatory responses in a variety of diseases including SLE. So we compared them with SRSR and analyzed their correlation with traditional markers such as erythrocyte sedimentation rate (ESR) and C‐reactive protein (CRP).

## 2 MATERIALS AND METHODS

### 2.1 Participants

This retrospective research collected medical information of 222 patients with SLE in the Department of Rheumatology and Immunology in the Second Affiliated Hospital of Chongqing Medical University (Chongqing, China) from January 2017 to April 2019; all of them were newly diagnosed and conformed to the classification diagnostic criteria of the American College of Rheumatology (ACR) in 1997.^4^ Patients with SLE are excluded from lymphoproliferative disease, hematopathy, blood transfusion, malignant tumor, other autoimmune diseases, infection, or liver diseases. At the same time, we selected 202 sex‐ and age‐matched healthy physical examiners during the same period in the same hospital as the control group; the Ethics Committee of the Second Affiliated Hospital of Chongqing Medical University approved the research program.

### 2.2 Date collection

By looking at the medical records, we extracted demographic characteristics, and laboratory and clinical data of entire participants in the hospital. The following indicators were collected from every subject: SOD, RDW‐SD, variation coefficient of erythrocyte distribution width (RDW‐CV), anti–double‐stranded DNA antibodies (anti‐dsDNA), white blood cell count (WBC), neutrophil count (Neu), red blood cell count (RBC), lymphocyte count (Lym), platelet (PLT), mean platelet volume (MPV), complement 3 (C3), ESR, CRP, and complement 4 (C4), and we calculated the value of NLR, SRSR, and PLR. In addition, we evaluated activity of disease through SLE Disease Activity Index (SLEDAI) score.^5^ The laboratory results were collected before treatment in all SLE patients.

### 2.3 Materials

Blood routine‐related indicators such as WBC, Neu, Lmy, RBC, PLT, and MPV were tested with Sysmex XN‐3000 and its supporting reagents; C3 and C4 were detected using automatic protein analyzers; ESR was determined by ESR‐30 dynamic monitor; anti‐dsDNA was detected by oumeng dot method; and SOD was detected by colorimetric method. Conduct daily indoor quality control before blood sample test. After passing the quality control, start the test according to the standard operating procedure of the instrument.

### 2.4 Statistical analysis

Statistical analysis of all the data used the program SPSS 19.0. If the data conform to the normal distribution, the statistics were described as mean ± standard deviation, and we compared those indicators between the two groups using the method of *t* test. If the data did not conform to the normal distribution, the median (M) and quartile (P25, P75) are used to describe the data, and we compared those indicators between the two groups through the Mann‐Whitney *U* test. Meanwhile, we compared the counting data between SLE group and control group by chi‐square test, Pearson's method was used to analyze the relationship between SRSR and other general inflammatory indicators, and the predictive value of SRSR in the patient group was determined through the ROC curve. In addition, we evaluated the relationship between SRSR, ESR, NLR, PLR, and SLEDAI by multiple linear regression analysis. If *P* < .05 in the tests, it would be considered that there was a statistical difference.

## 3 RESULTS

### 3.1 Baseline data of subjects

There was no statistical difference in sex, age, MPV, PDW, or Neu between SLE group and control group. The RBC, WBC, Hb, and lymphocyte counts in SLE patients were significantly lower than those in the control group (*P* < .05). In addition, serum SOD level and C4 were significantly reduced (*P* < .05), RDW‐SD, RDW‐CV, and anti‐dsDNA were higher than those of the control group (*P* < .05), and the clinical characteristics and laboratory examination results of 222 patients with SLE and 202 healthy people are shown in Table 1.

**Table 1:** Baseline characteristics of the participants

Characteristic	SLE patients (n = 222)		Healthy controls (n = 202)		*P*‐value
	n	Results	n	Results	
Sex (male/female)	222	17/205	202	21/172	.26
Age (years)	222	40 ± 15.68	202	44 ± 14.15	.10
SOD (U/mL)	222	108.95 (89.6,131.3)*	202	137.4 (127.5,148.2)	<.05
WBC (×10^9^/L)	222	4.81 (3.45,6.69)*	202	5.36 (4.36,7.66)	<.05
Neu (×10^9^/L)	222	3.47 (2.31,5.32)	202	3.285 (2.5,4.09)	.28
Lym (×10^9^/L)	222	1.045 (0.67,1.56)*	202	1.58 (1.26,2.09)	<0.05
RBC (×10^9^/L)	222	3.91 (3.26,4.42)*	202	4.4 (4.12,4.68)	<0.05
Hb (g/L)	222	112.5 (89,125)*	202	133 (125,140)	<.05
PLT (×10^9^/L)	222	167 (115,220)*	202	202 (169,241)	<.05
RDW‐CV (%)	222	14.4 (13.5,15.8)*	202	13.2 (12.7,13.8)	<.05
RDW‐SD	222	45.6 (42.2,50.8)*	202	43.35 (41.5,45.1)	<.05
PDW	200	13.55 (12.1,15.7)	202	13.6 (12.1,15.9)	.95
MPV (fl)	199	11 (10.3,11.8)	202	10.9 (10.3,11.8)	.66
C3 (g/L)	177	1.05 (0.79,1.30)*	150	1.36 (0.9,2.34)	<.05
C4 (g/L)	177	0.12 (0.06,0.21)*	150	0.35 (0.06,0.50)	<.05
Anti‐dsDNA（IU/mL）	182	67.7 (9.4,202.05)*	150	12.6 (7.43,47.38)	<.05
CRP (mg/dL)	172	8.68 (6.5,14.58)			
ESR (mm/h)	177	75 (38,90)			
SLEDAI	222	8 (6,11.25)			
Arthritis	222	104			
Alopecia	222	25			
Malar rash	222	152			

Note

Age is expressed by the mean ± SD and compared through two independent sample *t* tests, sex is expressed by the ratio between male and female and compared through chi‐square test, and other indicators were expressed by the median and quartile (P25, P75) and compared through the Mann‐Whitney *U* test.

Abbreviations: Anti‐dsDNA, anti–double‐stranded DNA antibodies; C3, complement 3; C4, complement 4; CRP, C‐reactive protein; ESR, erythrocyte sedimentation rate; Hb, hemoglobin; Lym, lymphocyte count; MPV, mean platelet volume; Neu, neutrophil count; PDW, platelet volume distribution width; PLT, platelet; RBC, red blood cell count; RDW‐CV, variation coefficient of erythrocyte distribution width; RDW‐SD, standard deviation of erythrocyte distribution width; SLEDAI, SLE Disease Activity Index; SOD, superoxide dismutase Dx; WBC, white blood cell count.

* *P*<.05.

### 3.2 Comparison of SRSR and other indicators between the two groups

**Figure 1:**
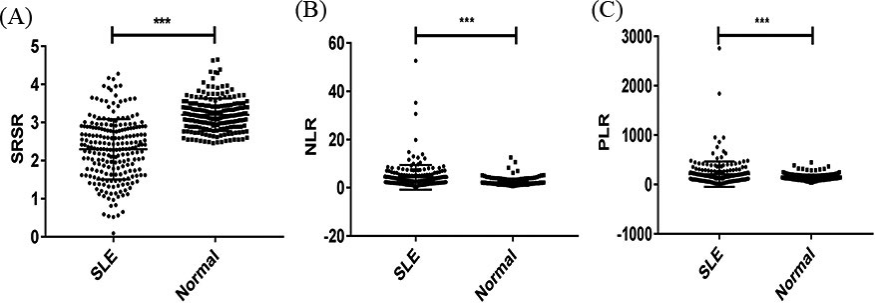
Comparison of SRSR, NLR, and PLR between SLE and control groups by scatter plot, ^***^ *P* < .05. A, shows the difference of SRSR in the two groups; B, shows the difference of NLR in the two groups; C, shows the difference of PLR in the two groups. NLR, neutrophil‐to‐lymphocyte ratio; PLR, platelet‐to‐lymphocyte ratio; SRSR, superoxide dismutase‐to‐standard deviation of erythrocyte distribution width ratio.

By comparing the NLR, PLR, and SRSR in SLE patients and the normal control group, it was found that the value of NLR and PLR in SLE patients was significantly higher than that in the normal control group (*P* < .05); however, SRSR in SLE patients was significantly lower than that in the normal control group, and the difference was statistically significant (*P* < .05). The results are shown in Figure 1.

### 3.3 Correlation analysis of SRSR and inflammatory indicators

**Figure 2:**
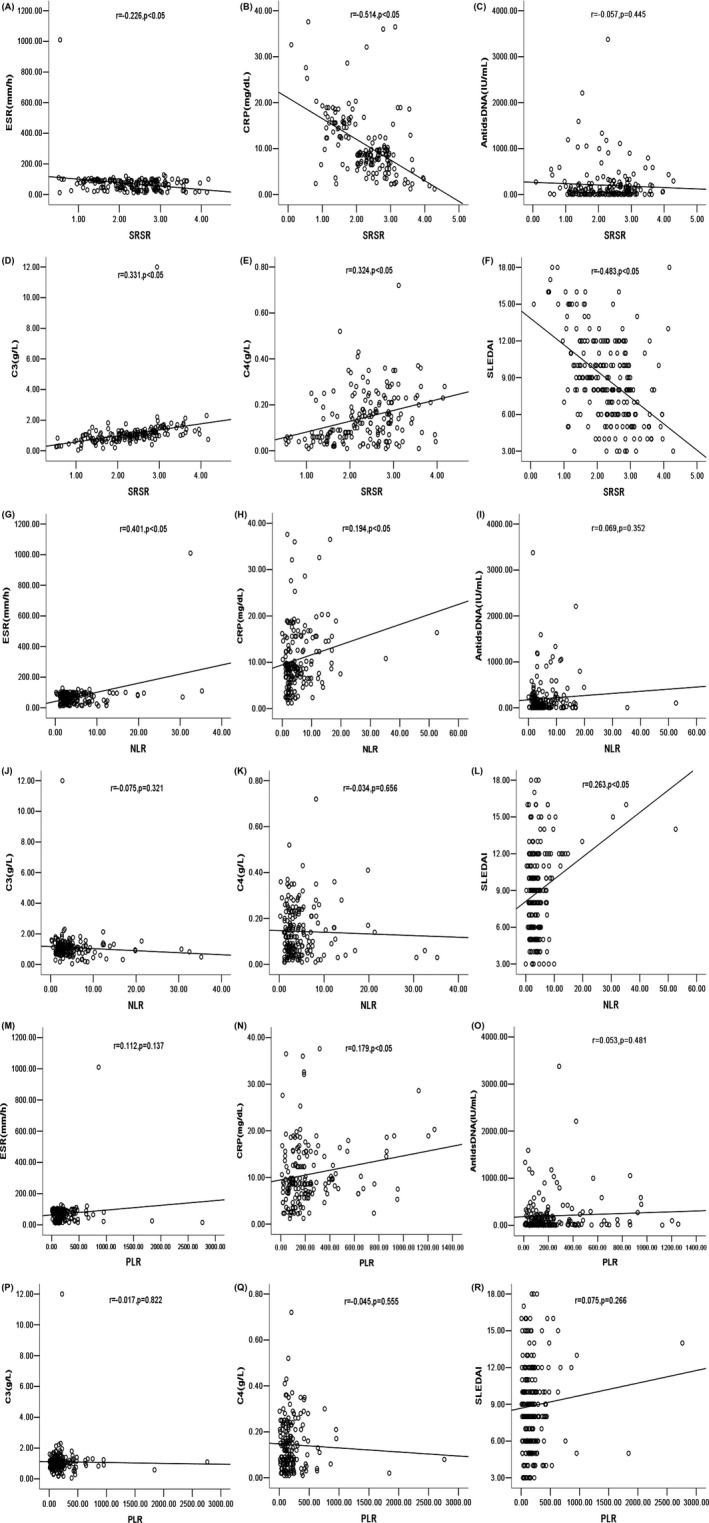
Correlation analysis and fitting curve. A, shows the correlation and curve between SRSR and ESR; B, shows the correlation and curve between SRSR and CRP; C, shows the correlation and curve between SRSR and anti‐dsDNA; D, shows the correlation and curve between SRSR and C3; E, shows the correlation and curve between SRSR and C4; F, shows the correlation and curve between SRSR and SLEDAI; G, shows the correlation and curve between NLR and ESR; H, shows the correlation and curve between NLR and CRP; I, shows the correlation and curve between NLR and anti‐dsDNA; J, shows the correlation and curve between NLR and C3; K, shows the correlation and curve between NLR and C4; L, shows the correlation and curve between NLR and SLEDAI; M, shows the correlation and curve between PLR and ESR; N, shows the correlation and curve between PLR and CRP; O, shows the correlation and curve between PLR and anti‐dsDNA; P, shows the correlation and curve between PLR and C3; Q, shows the correlation and curve between PLR and C4; R, shows the correlation and curve between PLR and SLEDAI; SRSR, superoxide dismutase‐to‐standard deviation of erythrocyte distribution width ratio; NLR, neutrophil‐to‐lymphocyte ratio; PLR: platelet‐to‐lymphocyte ratio. SLEDAI, SLE Disease Activity Index; C3, complement 3; C4, complement 4; ESR, erythrocyte sedimentation rate; CRP, C‐reactive protein; anti‐dsDNA, anti–double‐stranded DNA antibodies

According to Pearson's correlation analysis, it revealed a moderately negative correlation between serum SRSR and SLEDAI score in SLE patients (*r* = −.483, *P* < .05); there were a slight negative connection with ESR (*r* = −.226, *P* < .05), a slight positive connection with C3 (*r* = .331, *P* < .05), a slight positive correlation with C4 (*r* = .324, *P* < .05), a moderately negative correlation with CRP (*r* = −.514, *P* < .05), and no correlation with anti‐dsDNA (*r* = −.057, *P* = .445). In addition, there were a slight positive correlation between NLR and SLEDAI score (*r* = .263, *P* < .05), a slight positive connection with ESR (*r* = .401, *P* < .05), and no correlation between PLR and SLEDAI score (*r* = .075, *P* = .266). Thus, it could be seen that SRSR had a stronger correlation with disease activity in SLE than both NLR and PLR as shown in Figure 2.

### 3.4 SRSR is independently correlated with SLE disease activity

We analyzed the correlation between SRSR and SLE disease activity through multiple linear regression model after adjusting by the flowing factors: PLR, NLR, and SRSR, and taking the forward approach. The results are revealed in Table 2. After adjusting by NLR, it showed that SRSR was correlated with the SLEDAI score independently.

**Table 2:** Multiple linear regression analysis for the relationship between SLE and inflammatory marker

	B	Standard error	*P*
Constant	12.972	0.655	.000
SRSR	‐2.07	0.255	.000
NLR	0.156	0.040	.000

Abbreviations: NLR, neutrophil‐to‐lymphocyte ratio; SRSR, superoxide dismutase‐to‐standard deviation of erythrocyte distribution width ratio.

### 3.5 SRSR could predict the development of SLE

**Figure 3:**
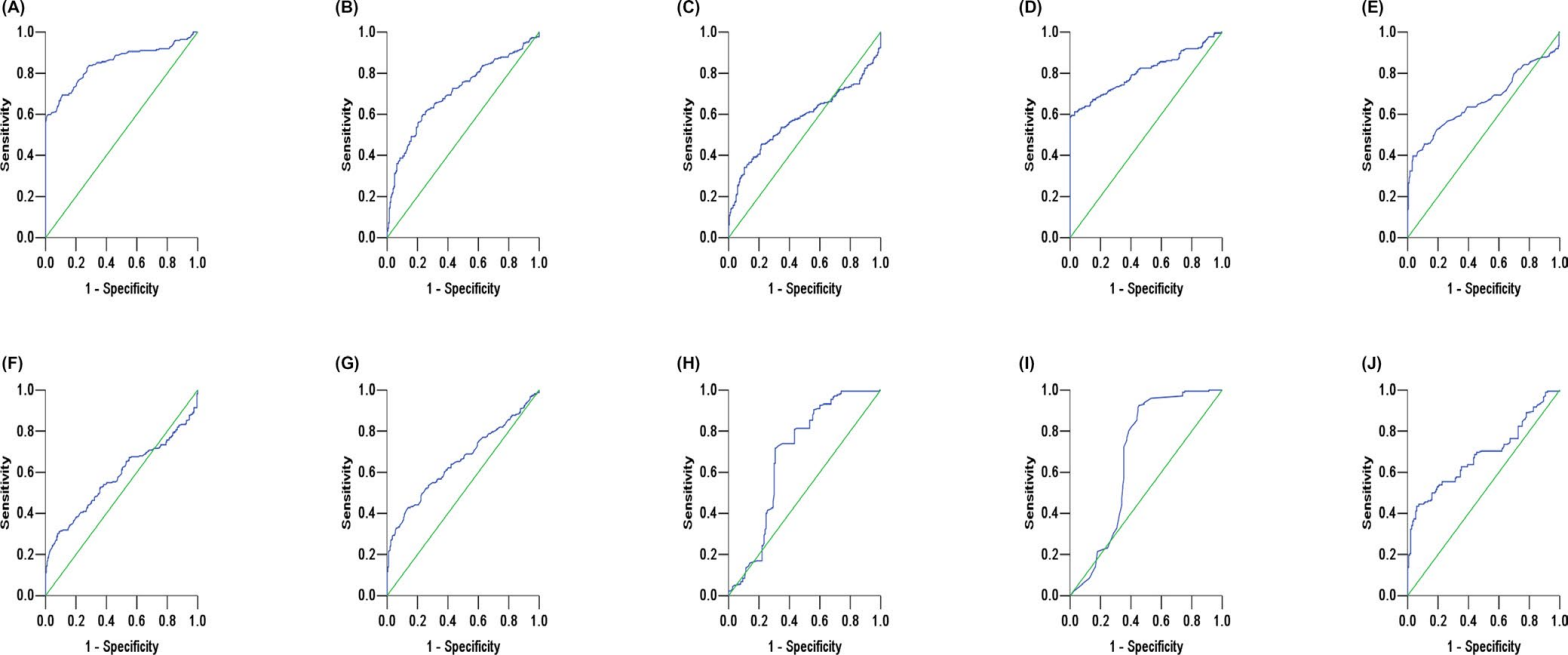
Receiver operating characteristic curve of indicators in SLE patients. A, shows the ROC curve of SRSR; B, shows the ROC curve of NLR; C, shows the ROC curve of PLR; D, shows the ROC curve of SOD; E, shows the ROC curve of RDW‐SD; F, shows the ROC curve of WBC; G, shows the ROC curve of PLT; H, shows the ROC curve of C3; I, shows the ROC curve of C4; J, shows the ROC curve of anti‐dsDNA. C3, complement 3; C4, complement 4; NLR, neutrophil‐to‐lymphocyte ratio; PLR, platelet‐to‐lymphocyte ratio; PLT, platelet; RDW‐SD, standard deviation of erythrocyte distribution width; SOD, superoxide dismutase; SRSR, superoxide dismutase‐to‐standard deviation of erythrocyte distribution width ratio; WBC, white blood cell count.

According to analyzing the ROC curve, it is found that the area under the ROC curve of SRSR was 0.849 (95% CI 0.811‐0.887). The AUC of NLR was 0.706 (95% CI 0.657‐0.756). The AUC of PLR was 0.577 (95% CI 0.522‐0.633). The AUC of serum SOD level was 0.807 (95% CI 0.764‐0.849). The AUC of RDW‐SD was 0.665 (95% CI 0.613‐0.718). The AUC of C3 was 0.683 (95% CI 0.621‐0.745). The AUC of C4 was 0.680 (95% CI 0.616‐0.744). The AUC of WBC was 0.579 (95% CI 0.525‐0.634). The AUC of PLT was 0.658 (95% CI 0.606‐0.709). The AUC of anti‐dsDNA was 0.684 (95% CI 0.628‐0.741). The AUC of SRSR was larger than that of SOD and other markers, suggesting that the diagnostic efficiency was stronger than that of SOD and RDW‐SD. The best cutoff value of SRSR for SLE was 2.68, the sensitivity was 0.685, and the specificity was 0.901; the best cutoff value of NLR for SLE was 2.68, the sensitivity was 0.60, and the specificity was 0.77; the best cutoff value of PLR for SLE was 203.17, the sensitivity was 0.342, and the specificity was 0.896. Therefore, SRSR is more sensitive and specific than NLR and PLR at the optimal cutoff value as shown in Figure 3.

## 4 DISCUSSION

Superoxide dismutase is a kind of active protease containing metal elements that exist widely in animals and plants. SOD1, SOD2, and SOD3 have been found in human cells. Fridovich et al^6^ demonstrated the role of SOD1 as a superoxide scavenger is a detoxifying metalase. It mainly exists in cytoplasmic, peroxidase, and mitochondrial membrane space, and SOD1 accounts for about 90% of SOD activity in eukaryotic cells.^7^ SOD is an important antioxidant enzyme in the organisms, which can catalyze the disproportionation reaction of superoxide anions to remove excessive oxygen free radicals in the body.^8^, ^9^ It plays an important part in the nosogenesis of SLE by participating in maintaining the balance of the oxidation‐antioxidant system in the body.^10^ RDW‐SD mainly refers to the variation degree of red blood cell volume in peripheral blood circulation, which is one of the routine examination items of whole blood cell. It is indicated that RDW is related to the inflammatory response of the body. Poz et al^11^ reported that there was strong relationship between RDW and the diagnosis and prognosis of vascular aging and cardiovascular diseases. Nakamura et al^12^ showed that when the inflammatory response of the body was activated, red blood cells were destroyed in large quantities, which promotes the hematopoietic production of bone marrow and accelerates the production of immature RBC. As a result, the volume of red blood cells in the peripheral blood is uneven, which can be shown as increased RDW‐SD. In autoimmune diseases, RDW‐SD was also used to evaluate the degree of inflammatory response.^13^ As can be seen from the above, both SOD and RDW‐SD play significant part in the pathogenesis of SLE. However, there are no literature reports which combine the SOD and RDW‐SD to reflect the activity and inflammation of SLE. In this research, SOD and RDW‐SD results of all subjects were collected to calculate the SOD‐to‐RDW‐SD ratio (SRSR), analyze its relationship with SLE activity and related inflammatory indicators, and explore the predictive value of SRSR for SLE.

In this study, we contrasted the results of WBC, RBC, PLT, RDW‐SD, and other blood routine tests between SLE group and normal control group, and then come to the conclusion that RBC, WBC, PLT, Neu, and Lym were all significantly reduced than those in the normal control group. From the above results, it is deduced that peripheral blood pancytopenia may exist in SLE patients to some extent, which is consistent with the changes of hematocytopenia in systemic inflammatory response. According to comparing with the control group, this research found that the SRSR level was significantly decreased in SLE patients (*P* < .05). Correlation analysis showed that SRSR level in SLE group was moderately negatively associated with SLEDAI score. In addition, after adjusting by other inflammatory markers, the regression analysis indicated that SRSR was associated with SLEDAI score independently. These findings show that SRSR may be a readily detectable and available laboratory parameter which can reflect the activity and inflammation in SLE patients. Our study also showed that there was no correlation between SRSR and anti‐dsDNA. This phenomenon may be caused by the medical intervention which could significantly affect the level of anti‐dsDNA or by the reason that anti‐dsDNA participates in the independent biological process of SLE.

It is well known that the composition of blood changes relatively in response to systemic inflammation, typically with neutropenia and lymphocytosis. In recent years, the level of Neu, Lym, and hemoglobin has been recognized as markers which can reflect the degree of phlegmonosis in many kinds of disease. As an indicator of phlegmonosis, NLR has been applied to reflect systemic inflammation in autoimmune diseases.^14^ Qin et al^14^ found that the value of NLR remarkably increased in SLE patients. Fawzy et al^15^ demonstrated that NLR significantly correlated with disease activity indices in recent‐onset RA patients thus reflecting systemic inflammation. In addition, PLR is also an inflammatory biomarker in many diseases, and the changes of PLR may be related to inflammation and cytokines. Mounting evidence showed that the level of PLR was associated with activity in patients with rheumatoid arthritis.^16^ Qin et al demonstrated that combining PLR with CA199 values may improve the diagnostic capacity of CA199 for PC in patients with type 2 diabetes. NLR combined with CA199 also resulted in improved diagnostic power, although the difference was not significant.^17^ It could be seen from the above that both NLR and PLR play an important part in reflecting clinical inflammation in SLE group. However, both of them are used to analyze their application value in SLE from the perspective of the body's inflammatory status. In this paper, the SOD, which is an antioxidant enzyme, and RDW‐SD, which can reflect the inflammatory state of the body, were combined through the ratio: SRSR, and then, we explored the relationship between SRSR and SLE. By comparing the results of NLR, PLR, and SRSR between SLE patients and control group, it was found that the three indexes had marked differences between the two groups. According to the analysis of correlation, it is indicated that SRSR has stronger correlation with SLEDAI than NLR and PLR. Our data implied that SRSR was more able to reflect subclinical inflammatory reaction and activity compared with PLR and NLR in SLE. Through the analysis of the area under the ROC curve, it showed that the AUC of SRSR was 0.849, the AUC of NLR was 0.706, and the AUC of PLR was 0.577. Among them, the AUC of SRSR was the highest. When the optimal cutoff value of SRSR was 2.68, the sensitivity was 0.685, and the specificity was 0.901. The sensitivity and specificity of SRSR were both stronger than NLR and PLR. In this study, the diagnostic efficacy of SOD and RDW‐SD for SLE alone was also analyzed, and we found that the AUC of SOD and RDW‐SD was smaller than SRSR, suggesting that SRSR has higher predictive value for SLE.

In summary, this study showed that SRSR was significantly reduced in SLE patients and that there was a certain correlation between SRSR and traditional inflammation‐related substances such as ESR, C3, and C4 in SLE patients. These results suggested that SRSR may be identified as an indicator which could be used to evaluating inflammation and activity in SLE and that it has high clinical application value. In addition, this article also has some imperfections. Firstly, it was a single‐center retrospective research, which cannot fully reflect the characteristics of the overall patients; more and larger multi‐center studies were needed to verify this result. Secondly, the specific mechanism of SRSR participating in SLE was still unclear, so further research was needed.
